# ‘Tug of war’ between competing phases in Li-rich cathodes revealed by analytical electron microscopy

**DOI:** 10.1093/nsr/nwaf288

**Published:** 2025-07-26

**Authors:** Shiyuan Zhou, Yuzi Liu, Khalil Amine, Gui-Liang Xu

**Affiliations:** Chemical Sciences and Engineering Division, Argonne National Laboratory, USA; Centre for Nanoscale Materials, Argonne National Laboratory, USA; Chemical Sciences and Engineering Division, Argonne National Laboratory, USA; Chemical Sciences and Engineering Division, Argonne National Laboratory, USA

Lithium-rich manganese-based oxides (LRMOs) have long been regarded as promising cathode materials for next-generation high-energy lithium-ion batteries (LIBs) [1–3]. Manganese, owing to its high natural abundance and low cost compared with nickel, presents a sustainable alternative for large-scale energy storage. LRMOs harness both cationic redox (from transition metals) and anionic redox (from lattice oxygen), enabling high capacities approaching 300 mA h g^–1^ at high operating voltages (>4.5 V vs. Li^+^/Li) [4,5]. As a result, LRMOs are positioned to bridge the energy-density gap between conventional LIBs—such as Ni-rich layered oxide cathodes, typically limited to ∼220 mA h g^–1^—and future high-capacity systems such as lithium sulphur and lithium oxygen batteries, which face critical challenges related to insulating discharge products and intermediate shuttling effects. Despite extensive efforts in surface engineering and electrolyte optimization, the intrinsic structure property of LRMOs remains unresolved. A main debate persists over whether these materials form a chemically mixed one-phase solid solution (e.g. Li_1+_*_x_*Ni*_y_*Co*_z_*Mn_1–_*_x_*_–_*_y_*_–_*_z_*O_2_) or consist of a nanoscale two-phase mixture comprising monoclinic Li_2_MnO_3_ and layered LiMO_2_ (M represents transition metals, such as Ni, Mn and Co) [6–9]. This uncertainty significantly impedes the rational design of LRMOs for stable cycling and high-rate performance.

**Figure 1.**
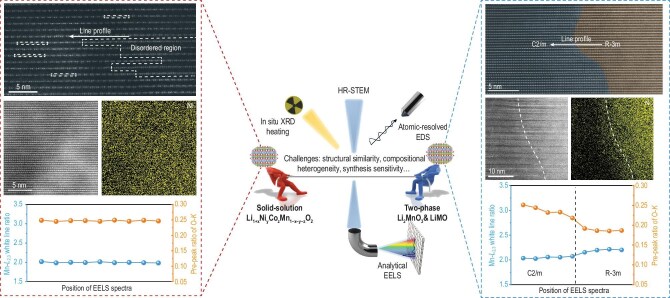
‘Tug of war’ between different structures in LRMOs. The schematic illustrates the competing structural models of LRMOs—solid-solution (Li_1+_*_x_*Ni*_y_*Co*_z_*Mn_1–_*_x_*_–_*_y_*_–_*_z_*O_2_) versus chemically separated two-phase (CSTP, Li_2_MnO_3_ and LiMO_2_)—and the analytical techniques used to resolve their distinction. On the left, HR-STEM, Ni mapping and uniform electron energy loss spectroscopy white-line and oxygen pre-peak ratios support a solid-solution structure. On the right, clear phase boundaries between *C*2/*m* and *R*${\mathrm{\bar{3}}}$*m* domains, revealed by using atomic-resolution imaging and Ni segregation, correspond to a CSTP structure. Figures were reproduced from [10] with permission.

Precisely determining the underlying structure of LRMOs remains a significant challenge. Structurally, these materials are highly sensitive to various synthesis parameters—such as chemical composition, sintering temperature and cooling rate—all of which can determine whether a one-phase solid solution or a two-phase coexistence emerges. From the characterization part, powder X-ray diffraction (XRD) and neutron diffraction are commonly used to index different phases. Aberration-corrected scanning transmission electron microscopy (STEM) can offer atomic-scale insights into local structures. However, both approaches face critical limitations in resolving phase distinctions. First, the structural similarity between monoclinic (*C*2/*m*) and rhombohedral (*R*${\mathrm{\bar{3}}}$*m*) phases leads to overlapping diffraction patterns. For example, LRMOs have been indexed to both phases, with differentiation relying on subtle features such as superlattice reflections between 20° and 30° of the monoclinic phase (*C*2/*m*) [8]. Second, structural heterogeneity—both interparticle and intraparticle—adds complexity. STEM analyses are conducted at localized sites, making it difficult to distinguish the phase coexistence within individual particles, let alone the similar lattice contrast of different phases. As a result, it remains unclear whether the diversity in reported structures reflects intrinsic differences induced by synthesis conditions or artefacts arising from the characterization methods. Moreover, the presence of overlapping lattice modulations, stacking faults and slight orientation variations further obscures the distinction between true phase separation and a modulated solid solution. These factors together contribute to the ongoing ‘tug of war’ over the precise phase identity in LRMOs (Fig. 1).

The formation of two distinct phases—LiMO_2_ and Li_2_MnO_3_—is highly sensitive to synthetic conditions, such as temperature-dependent phase transition. However, accurately identifying and distinguishing these phases remain challenging, especially in resolving structures across different length scales and spatial depths, from the bulk to the surface. The recent work by Su and co-workers provides valuable new insight by systematically tracing the thermal evolution of Li_1.2_Ni_0.2_Mn_0.6_O_2_ by using a comprehensive set of techniques that span atomic to bulk scales [10]. By combining *in situ* heating XRD, high-resolution STEM, atomic-resolved energy-dispersive X-ray spectroscopy, analytical electron energy loss spectroscopy and thermodynamic modelling, they revealed a temperature-dependent structural transformation. Specifically, it was found that the nanosized phases transition into a solid-solution structure (*C*2/*m*) when the temperature was increased from 550°C to 800°C, and further grew larger up to 850°C. However, annealing at 900°C induces a pronounced transition into a chemically separated two-phase (CSTP) structure composed of Li_2_MnO_3_ (*C*2/*m*) and LiNi_0.5_Mn_0.5_O_2_ (*R*${\mathrm{\bar{3}}}$*m*). The CSTP structure exhibits clear compositional separation and coherent crystallographic interfaces, as visualized by using analytical STEM. This phase segregation is thermodynamically driven, facilitated by Ni^2+^ migration and oxygen vacancy formation at elevated temperatures. Importantly, the emergence of the CSTP structure correlates with degraded electrochemical performance compared with the solid-solution counterpart, implying the trade-offs introduced by high-temperature phase segregation. This finding emphasizes the importance of controlled synthesis conditions to avoid undesirable phase separation and to preserve the reversible redox behaviour.

This study establishes a valuable framework for understanding and controlling phase evolution in LRMOs cathodes. The identification of temperature-triggered CSTP formation stresses the necessity for precise synthetic tuning to stabilize the desired structure. Looking forward, extending this approach to investigate phase-dependent electrochemical reaction mechanisms under operating conditions could yield deeper insights into the interplay between oxygen redox activity, ion migration and structural stability. By bridging long-standing structural ambiguities, this work represents a significant step toward the rational design of high-capacity, high-stability LRMOs cathode materials.
